# Simultaneous Insertion of Two Ligands in gD for Cultivation of Oncolytic Herpes Simplex Viruses in Noncancer Cells and Retargeting to Cancer Receptors

**DOI:** 10.1128/JVI.02132-17

**Published:** 2018-02-26

**Authors:** Valerio Leoni, Biljana Petrovic, Tatiana Gianni, Valentina Gatta, Gabriella Campadelli-Fiume

**Affiliations:** a Department of Experimental, Diagnostic and Specialty Medicine, University of Bologna, Bologna, Italy; b Nouscom SRL, Rome, Italy; University of California, Irvine

**Keywords:** HER2, HSV, retargeting, gD, Vero, oncolytic virus

## Abstract

Insertion of a single-chain variable-fragment antibody (scFv) to HER2 (human epidermal growth factor receptor 2) in gD, gH, or gB gives rise to herpes simplex viruses (HSVs) specifically retargeted to HER2-positive cancer cells, hence to highly specific nonattenuated oncolytic agents. Clinical-grade virus production cannot rely on cancer cells. Recently, we developed a double-retargeting strategy whereby gH carries the GCN4 peptide for retargeting to the noncancer producer Vero-GCN4R cell line and gD carries the scFv to HER2 for cancer retargeting. Here, we engineered double-retargeted recombinants, which carry both the GCN4 peptide and the scFv to HER2 in gD. Novel, more-advantageous detargeting strategies were devised so as to optimize the cultivation of the double-retargeted recombinants. Nectin1 detargeting was achieved by deletion of amino acids (aa) 35 to 39, 214 to 223, or 219 to 223 and replacement of the deleted sequences with one of the two ligands. The last two deletions were not attempted before. All recombinants exhibited the double retargeting to HER2 and to the Vero-GCN4R cells, as well as detargeting from the natural receptors HVEM and nectin1. Of note, some recombinants grew to higher yields than others. The best-performing recombinants carried a gD deletion as small as 5 amino acids and grew to titers similar to those exhibited by the singly retargeted R-LM113 and by the nonretargeted R-LM5. This study shows that double retargeting through insertion of two ligands in gD is feasible and, when combined with appropriate detargeting modifications, can result in recombinants highly effective *in vitro* and *in vivo*.

**IMPORTANCE** There is increasing interest in oncolytic viruses following the FDA and European Medicines Agency (EMA) approval of the oncolytic HSV Oncovex^GM-CSF^ and, mainly, because they greatly boost the immune response to the tumor and can be combined with immunotherapeutic agents, particularly immune checkpoint inhibitors. A strategy to gain high cancer specificity and avoid virus attenuation is to retarget the virus tropism to cancer-specific receptors of choice. However, cultivation of retargeted oncolytics in cells expressing the cancer receptor may not be approvable by regulatory agencies. We devised a strategy for their cultivation in noncancer cells. Here, we describe a double-retargeting strategy, based on the simultaneous insertion of two ligands in gD, one for retargeting to a producer, universal Vero cell derivative and one for retargeting to the HER2 cancer receptor. These insertions were combined with novel, minimally disadvantageous detargeting modifications. The current and accompanying studies indicate how to best achieve the clinical-grade cultivation of retargeted oncolytics.

## INTRODUCTION

Oncolytic viruses have come of age (1–5) since the approval by the FDA and the European Medicines Agency (EMA) of an oncolytic herpes simplex virus (HSV), initially named Oncovex^GM-CSF^ or T-Vec, for the treatment of metastatic melanoma (6, 7). Several generations of oncolytic HSVs were designed and tested in preclinical assays and in clinical trials. Many of them achieve cancer specificity by virtue of attenuation, frequently obtained through the deletion of the γ_1_34.5 gene, whose product counteracts the interferon (IFN) and protein kinase R (PKR) response of the cell to the virus (7–10). In Oncovex^GM-CSF^ and other examples, additional genes were deleted (11, 12). The resulting recombinants exhibited various degrees of attenuation. A drawback of attenuation is that not all cancer cells sustain a robust replication of these viruses.

An alternative strategy to attenuation has been to obtain cancer specificity through the modification of the HSV tropism and tropism retargeting to a cancer-specific receptor of choice, coupled with detargeting from natural receptors (13–21). In our laboratory, the targeted cancer receptor is human epidermal growth factor receptor 2 (HER2), expressed in breast, ovary, stomach, lung, and other cancers (22). While the HER2-positive cancers are usually treated with anti-HER2 monoclonal antibodies, exemplified by trastuzumab and pertuzumab, only a fraction of cancers are sensitive to this treatment, and resistance develops frequently (23).

HSV enters cells through the concerted action of four envelope glycoproteins, named gD, gH/gL, and gB, which are activated in a cascade fashion by interaction with cognate receptors and intermolecular signaling (24–29). Briefly, gD interacts alternatively with herpesvirus entry mediator (HVEM) or nectin1 (30–32). The receptor-bound gD activates gH/gL, which is additionally activated by αvβ6 or αvβ8-integrins (33, 34). gH activation results in the displacement of gL (35) and is then transmitted to gB, which executes the fusion between the virion envelope and the cell membrane (36). In the retargeted viruses, a new ligand, exemplified by a single-chain variable-fragment antibody (scFv) to HER2, is engineered in gD, in gH, or in gB, while appropriate deletions in gD ensure the detargeting from gD natural receptors (13, 15–17, 37, 38). The chimeric glycoproteins that carry the scFv to HER2 mediate HSV entry through HER2. Because of the detargeting-retargeting process, these oncolytic HSVs depend strictly on HER2 for infection.

For clinical-grade preparations of retargeted oncolytic HSVs, it is advisable to avoid virus cultivation in HER2-positive cancer cells. To meet these needs, we recently developed a system for the cultivation in noncancer cells of HSVs retargeted to HER2 and, potentially, to any cancer-specific receptor of choice. The system is based on a double-retargeting strategy. One retargeting is to the HER2 or any cancer receptor of choice. The other retargeting is by way of the 20-amino-acid (aa)-long GCN4 peptide, which readdresses the tropism to Vero cells expressing the artificial receptor named GCN4R (39). The latter is made by a single-chain antibody to GCN4 (40) fused to domains II, III, TM, and C tail of nectin1. The choice of the Vero cells as recipients of GCN4R rested on the notion that wild-type (wt) Vero cells have been approved by the FDA for the clinical-grade preparations of Oncovex^GM-CSF^ (where GM-CSF is granulocyte-macrophage colony-stimulating factor; the commercial name is Imlygic), the derivative named Vero-His is approved for clinical-grade preparations of oncolytic measles viruses (41), and more generally, wt Vero cells are approved for growth of a number of human vaccines. The R-213 recombinant was readdressed to GCN4R by engineering the GCN4 peptide in gH; simultaneously, it was readdressed to HER2 by insertion of the scFv to HER2 in gD, in place of aa 6 to 38 (39). This deletion detargets HSV tropism from HVEM and nectin1 (17).

The aims of this work were 2-fold: first, to explore alternative ways to coexpress the scFv to HER2 for cancer retargeting and the GCN4 peptide for *in vitro* cultivation in the Vero-GCN4R cells; second, to define novel, less disadvantageous detargeting strategies so as to optimize the cultivation of retargeted oncolytic HSVs in the noncancer cells.

## RESULTS

### Double gD retargeting and novel detargeting.

An aim of this work was to ascertain whether gD can simultaneously accept two retargeting moieties, the GCN4 peptide and the scFv to HER2. To better accomplish this task, we reduced the size of the deletion in gD, so as to maintain the detargeted phenotype and preserve gD sequences and possibly the gD structure as much as possible. Our initial gD detargeted/retargeted viruses R-LM113 and R-LM249, which carry the deletions of aa 6 to 38 (Δ6–38) and 61 to 218 (Δ61–218) (17, 19), respectively, were designed at times when the regions of interaction between gD and its receptors were known mainly through molecular biology approaches and through structural information on HVEM-bound gD (32). Indeed, the deletion of aa 38 in R-LM113 preceded the detailed knowledge of the nectin1 binding site in gD. Here, we took advantage of the information on gD contact area with nectin1, inferred from the structure of gD bound to nectin1, as determined by X-ray crystallography (42). According to the cocrystal structure, a tip in nectin1 protrudes into a groove in gD, whose critical residues include the previously known Y38 and the adjacent residues, including H39, and residues 215 and 220 to 223. Those structural studies suggested two alternative possibilities for nectin1 detargeting. One was the deletion of aa 35 to 39. The other was the deletion of the region that includes aa 214 to 223 (42), not assayed before in detargeting studies. Here we removed aa 214 to 223 or 219 to 223. The HVEM detargeting was achieved by the simple insertion of the GCN4 peptide or of the scFv to HER2 between aa residues 24 and 25, which are part of the HVEM binding site (32).

**FIG 1:**
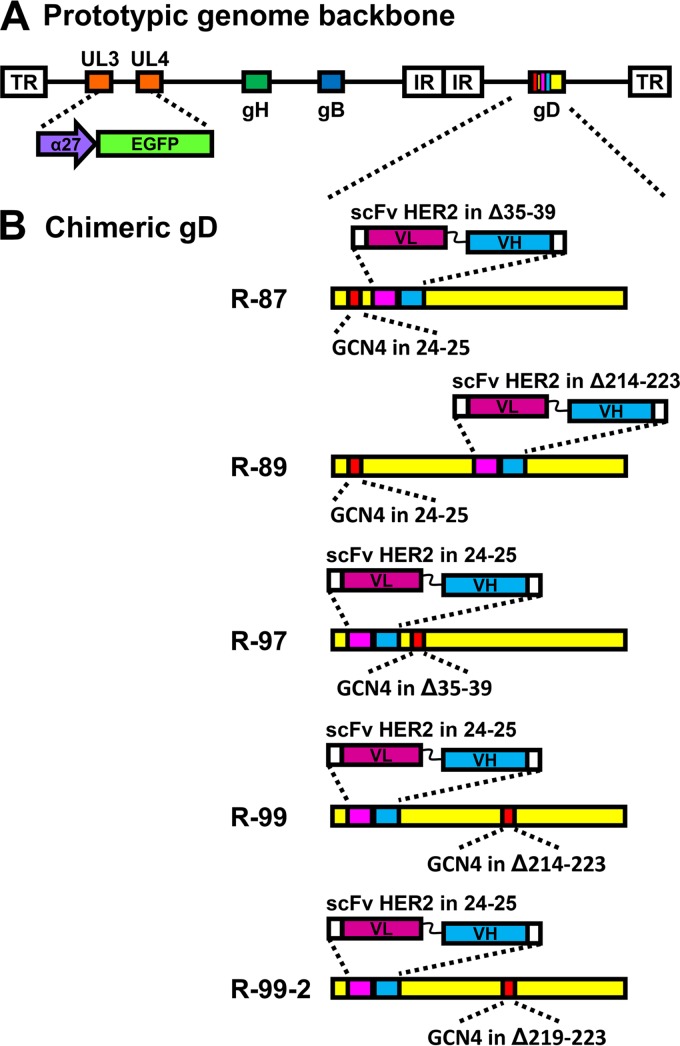
Genome arrangement of the recombinants generated in this study. (A) Prototypic genome arrangement of recombinants. Each recombinant carries the BAC sequence and the α27-promoter driven EGFP (enhanced green fluorescence protein), bracketed by LoxP sites, cloned in the UL3 and UL4 intergenic region, the GCN4 peptide, and the scFv to HER2 in appropriate sites of gD as detailed below. The unique long (UL) and unique short (US) portions of the genome, bracketed by terminal (TR) and internal repeats (IR), along with the location of gB and gH genes, are shown. (B) Specific genotypic modifications in the gD gene of each recombinant.

**FIG 2:**
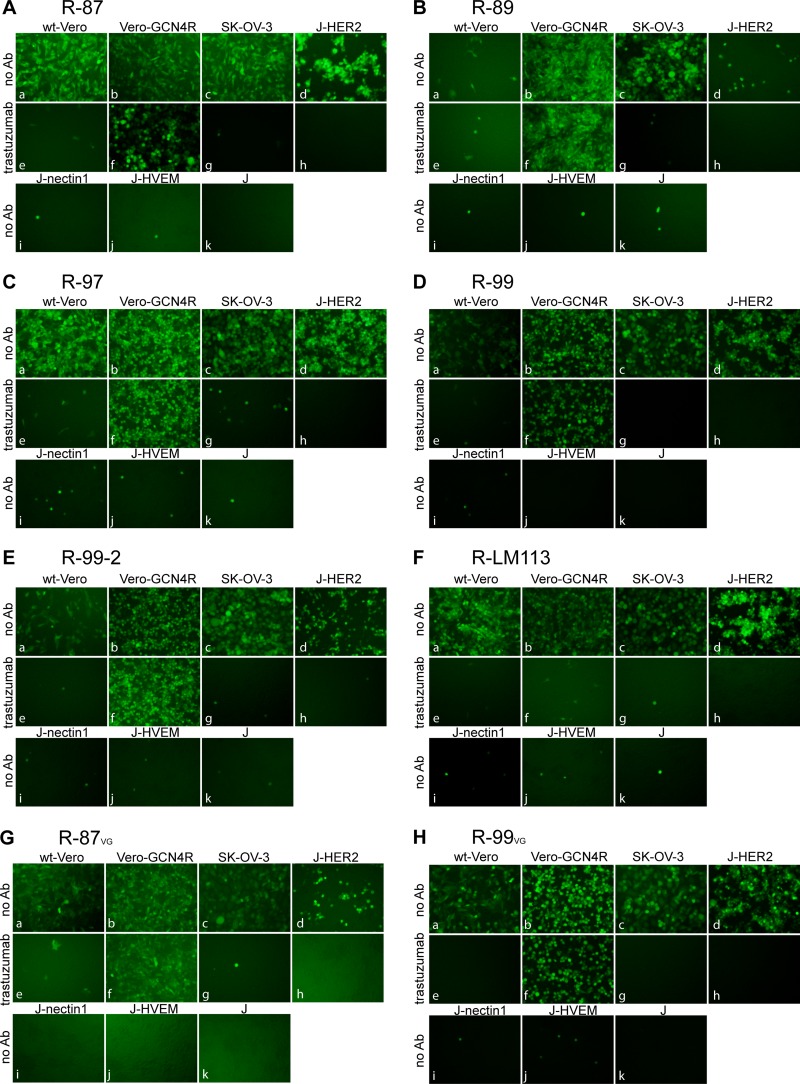
Tropism of R-87, R-89, R-97, R-99, and R-99-2 recombinants and, for comparison, of R-LM113 in the indicated cell lines. (A to F) The indicated cells were infected with R-87 (A), R-89 (B), R-97 (C), R-99 (D), R-99-2 (E), and for comparison, R-LM113 (F) at a multiplicity of infection (MOI) of 1 PFU/cell and monitored for EGFP expression by fluorescence microscopy 24 h postinfection. J cells express no receptor for wt HSV; J-HER2, J-nectin1, and J-HVEM express the indicated receptor. Infection was carried out in the absence of antibodies (no Ab) or in the presence of the humanized anti-HER2 monoclonal antibody trastuzumab at a concentration of 28 μg/ml. (G, H) Tropism of R-87 (G) and R-99 (H) recombinants grown in Vero-GCN4R cell. Cells were infected and monitored for EGFP expression as described above. The panels were adjusted by means of Adobe Photoshop software to match one to the other in the final gallery. The level, brightness, and contrast of each panel were adjusted as follows: R-87 (A) panels a, b, c, f, + 35 + 75 + 100; panels d, e, g, h, i, j, k, + 35 + 25 + 100; R-89 (B) panels a, b, e, f, + 35 + 75 + 100; panels c, g, + 35 + 75 0; panels d, h, i, j, k, + 35 + 25 + 100; R-97 (C) panels a, b, e, f, g, + 35 + 75 + 100; panel c, + 35 + 75 0; panels d, h, + 35 + 25 0; panels i, j, k, + 35 + 25 + 100; R-99 (D) panels a, c, g, + 35 + 75 0; panels b, e, f, + 35 + 75 + 100; panels d, h, + 35 + 25 0; panels i, j, k, + 35 + 25 + 100; R-99-2 (E) panels a, b, e, f, + 35 + 75 + 100; panels c, g, + 35 + 75 0; panels d, h, i, j, k, + 35 + 25 + 100; R-LM113 (F) panels a, b, e, f, + 35 + 75 + 100; panels c, g, + 35 + 75 0; panels d, h, i, j, k, + 35 + 25 + 100; R-87_VG_ (G) panels a, b, + 50 + 75 + 100; panel c, + 35 + 100 + 0; panels d, e, f, g, + 50 + 25 + 100; panels h, i, j, k, + 50 0 + 100; R-99_VG_ (H) panels a, b, e, f, + 35 + 75 + 100; panel c, + 35 + 75 0; panels d, h, i, j, k, + 35 + 25 + 100; panel g, + 15 + 75 0.

The list of double-insertion gD recombinants is reported in Table 1, which also summarizes the essential phenotypic features of the recombinants. The genome backbone is shown in Fig. 1A. The specific genotypes are shown in Fig. 1B. A description of the viruses is given in European patent application PCT/EP2017/063948 (V. Leoni and M. G. Campadelli, 14 December 2017). The tropism was assayed in the HER2-positive cancer cells SK-OV-3, in wt Vero cells, and in Vero-GCN4R, which express the artificial receptor to GCN4 peptide (39), and in J cell derivatives. J cells express no receptor for HSV; the derivatives expressing a single receptor—HER2, nectin1, and HVEM—have been described previously (16, 43). R-LM113, retargeted to HER2 but not to GCN4R, was included as a control. The tropism of R-87, R-89, R-97, R-99, and R-99-2 is shown in Fig. 2A to F. Cumulatively, the results show the following. (i) All recombinants were detargeted from HVEM and from nectin1, since they failed to infect J-nectin1 and J-HVEM cells. (ii) All recombinants were retargeted to HER2, as inferred by the infection of J-HER2 and SK-OV-3 cells and by inhibition of infection by trastuzumab, the monoclonal antibody (MAb) to HER2 from which the scFv employed for retargeting was derived (44). This property is shared with R-LM113. (iii) All recombinants, except R-89, infected wt Vero cells. This infection was inhibited by trastuzumab; hence, it most likely occurred through the simian ortholog of human HER2 (hHER2). The genome sequence of the Vero cell is incomplete, and so far, there is no documentation of a HER2 homologue. However, Vero cells were isolated from an African green monkey (Chlorocebus sp.), and the sequence of the Chlorocebus genome contains the HER2 homologue (Chlorocebus sabaeus; RefSeq accession number XM_008012845.1) with 98% identity with the human HER2 at the amino acid level. (iv) All recombinants infected Vero-GCN4R cells. This infection was only in part decreased by trastuzumab, indicating that it occurred in part through the GCN4 peptide present in the recombinants and its interaction with the GCN4R. (v) There was no difference in the recombinant tropism whether the viruses were grown in Vero-GCN4R or in SK-OV-3 cells, as exemplified for R-87 and R-99 (Fig. 2G and H). Altogether, the results indicate that double retargeting through the insertion of two different retargeting moieties in gD is feasible. All three nectin1-detargeting strategies based on Δ35–39, Δ214–223, or Δ219–223 were effective. The detargeting through deletion of aa 214 to 223 or aa 219 to 223 regions had not been attempted before.

**TABLE 1:** Summary of genotypes and major phenotypic properties of the listed recombinants

Recombinant HSV-1	GCN4 position in gD	scFv-HER2 position in gD	Retargeting to HER2	Detargeting from nectin1/HVEM	Reference or source
R-87	24–25	Δ35–39	+	+	This paper
R-89	24–25	Δ214–223	+	+	This paper
R-97	Δ35–39	24–25	+	+	This paper
R-99	Δ214–223	24–25	+	+	This paper
R-99-2	Δ219–223	24–25	+	+	This paper
R-LM113	None	Δ6–38	+	+	17
R-LM5	None	No scFv, no deletion	−	−	17

### Comparative growth of double gD-retargeted recombinants.

**FIG 3:**
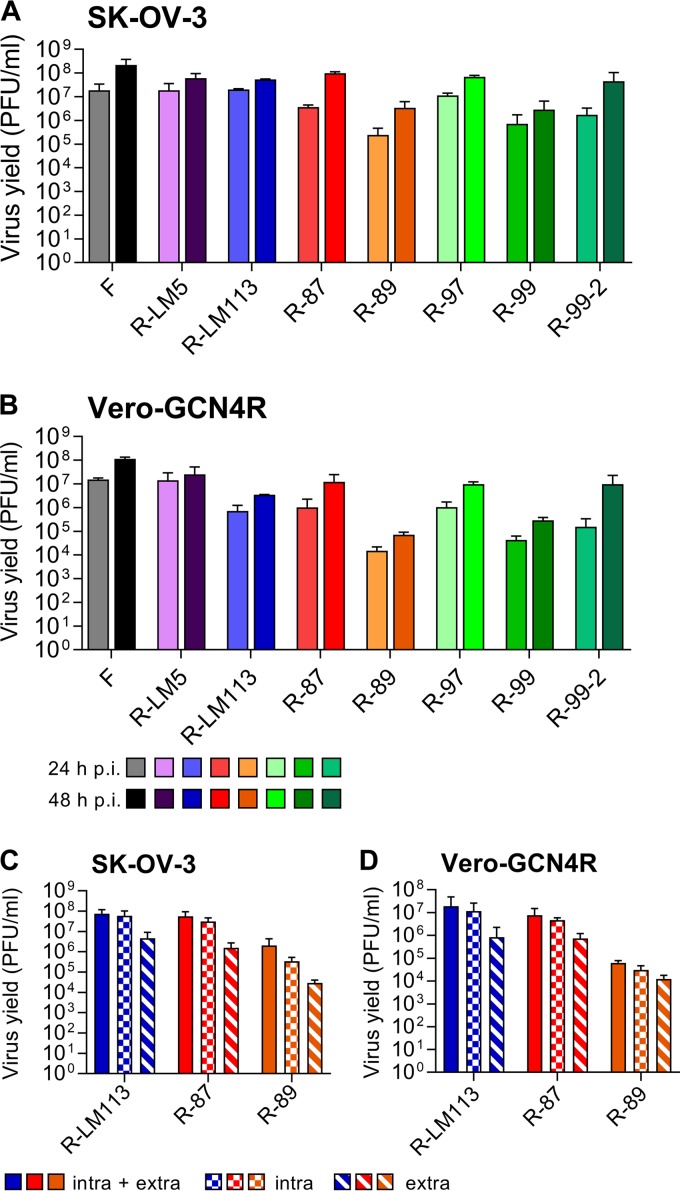
Yields of R-87, R-89, R-97, R-99, and R-99-2 recombinants and of R-LM5, R-LM113, and wt-HSV-1(F), for comparison. (A, B) SK-OV-3 (A) and Vero-GCN4R (B) cells were infected with the indicated viruses at 0.1 PFU/cell. Progeny virus collected at 24 or 48 h after infection was titrated in SK-OV-3 cells. Results represent the averages for triplicates ± SD. (C, D) Production of intracellular and extracellular R-87, R-89, and R-LM113 in SK-OV-3 (C) and in Vero-GCN4R (D) cells. Replicate cultures were infected as above. At 48 h after infection, medium (extra) and cells (intra) were harvested separately or together (intra + extra). Progeny virus was titrated in SK-OV-3 cells. Results represent the averages for triplicates ± SD.

We compared the yields of the above recombinants to those of the wt HSV-1(F), the wt-gD recombinant named R-LM5, and the singly HER2-retargeted R-LM113 in SK-OV-3 and in Vero-GCN4R cells. R-LM5 carries a wt gD, the bacterial artificial chromosome (BAC) plus enhanced green fluorescent protein (EGFP) sequences, and is therefore the wt counterpart of the retargeted HSVs. A representative experiment (Fig. 3A and B) shows that at 48 h after infection the yield of the recombinants R-87, R-97, and R-99-2 did not significantly differ one from the other, either in SK-OV-3 or in Vero-GCN4R cells. We note that R-LM113 replicated for one passage in wt Vero cells and their Vero-GCN4R derivative; however, numerous efforts to passage serially R-LM113 in these cells were unsuccessful and did not yield any progeny. The two recombinants with lower yields were R-89 and R-99. R-87 and R-89, representative of the best-performing and least well performing recombinants, respectively, were further analyzed with respect to the extent of virus release in the extracellular medium. Figure 3C and D shows that for both viruses the extracellular virus yield at 48 h was about 1 log lower than the intracellular virus yield, as was the case for R-LM113.

**FIG 4:**
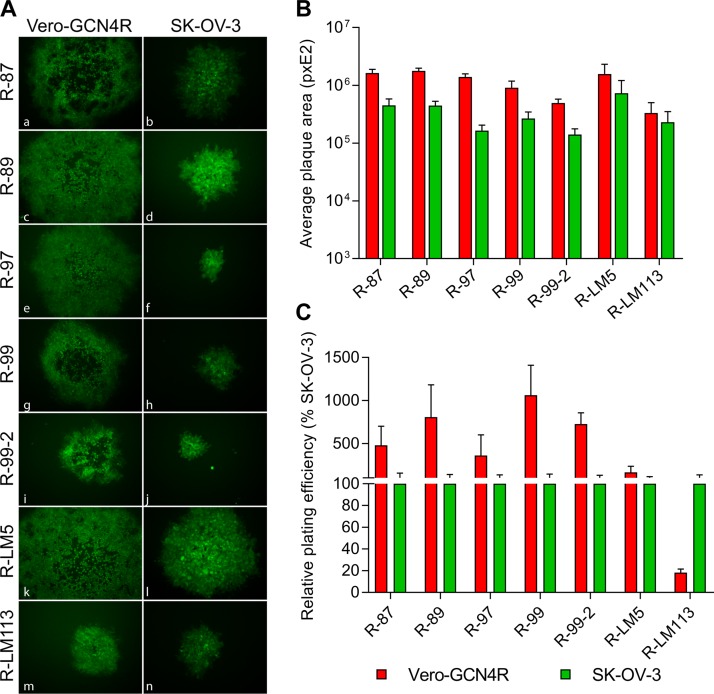
Plating efficiency of the indicated recombinants in Vero-GCN4R and SK-OV-3 cells. (A) A typical plaque is shown for each virus in the indicated cells. The subpanels were adjusted by means of Adobe Photoshop software to match one to the other in the final gallery. The level, brightness, and contrast of each panel were adjusted as follows. Panel a, + 30 + 50 + 100; panels b, h, n, + 20 + 50 + 100; panels c, d, k, l, + 50 + 25 + 50; panels e, f, + 35 + 20 + 100; panel g, + 35 + 50 + 100; panels i, j, + 35 + 25 + 100; panel m, + 30 + 75 + 100. (B) Average plaque size of the indicated recombinants in Vero-GCN4R and SK-OV-3 cells. Six pictures were taken for each recombinant. Plaque areas were measured with Nis Elements-Imaging software (Nikon). (C) Replicate aliquots of viruses were plated in SK-OV-3 and Vero-GCN4R cells. Plaques were scored 3 days later. The relative number of plaques formed by each virus in the indicated cell line is reported as the percentage of the number of plaques formed in SK-OV-3 cells. Results represent the average for triplicates ± SD.

Next, we analyzed the ability of the recombinants to form plaques, with respect to plaque size and plating efficiency. Figure 4A shows a typical plaque for each recombinant in Vero-GCN4R and SK-OV-3 cells. The average plaque size of the recombinants is shown in Fig. 4B. All recombinants formed somewhat larger plaques in Vero-GCN4R than in SK-OV-3 cells. Also, with respect to the number of plaques, the efficiency was somewhat higher in the Vero-GCN4R than in the SK-OV-3 cells (Fig. 4C). Altogether, these results show that the Vero-GCN4R cell line enables the efficient spread of the recombinants.

**FIG 5:**
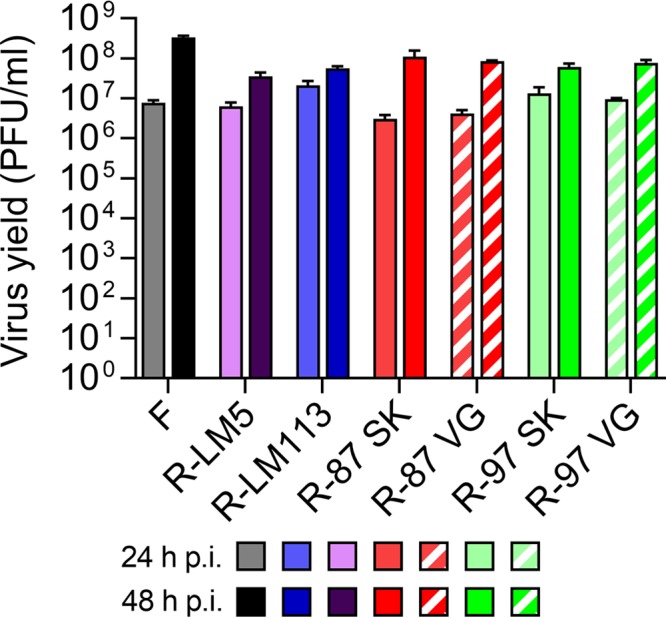
Comparative yields of R-87 and R-97 precultivated in SK-OV-3 or Vero-GCN4R cells. R-87 and R-97 were cultivated in SK-OV-3 (R-87_SK_, R-97_SK_) or in Vero-GCN4R (R-87_VG_, R-97_VG_) cells and employed to infect SK-OV-3 cells at 0.1 PFU/cell. Progeny virus harvested at 24 or 48 h after infection was titrated in SK-OV-3 cells. Results represent the averages for triplicates ± SD.

In the past, we observed that switching a virus from one cell line to a different cell line for replication may sometimes result in a lower replication rate at the earliest passages after the switch. Specifically, when a virus is grown in a certain cell line (e.g., Vero-GCN4R) and is then switched to another cell line (e.g., SK-OV-3), there may be a decrease in the efficiency of virus growth at very early passages. We analyzed whether the growth of R-87 and R-97 in Vero-GCN4R cells may affect the extent of replication in the cancer SK-OV-3 cells. R-87 and R-97 were grown in Vero-GCN4R (R-87_VG_ and R-97_VG_) or in SK-OV-3 cells (R-87_SK_ and R-97_SK_) and then employed to infect SK-OV-3 cells. Figure 5 shows that R-87_VG_ grew as efficiently as R-87_SK_ in SK-OV-3 cells. Similarly, R-97_VG_ grew as efficiently as R-97_SK_ in SK-OV-3 cells. Thus, switching from Vero-GCN4R to SK-OV-3 cells exerted no detrimental effect on the efficiency of viral growth.

### Cell-killing ability of double-gD-retargeted recombinants.

**FIG 6:**
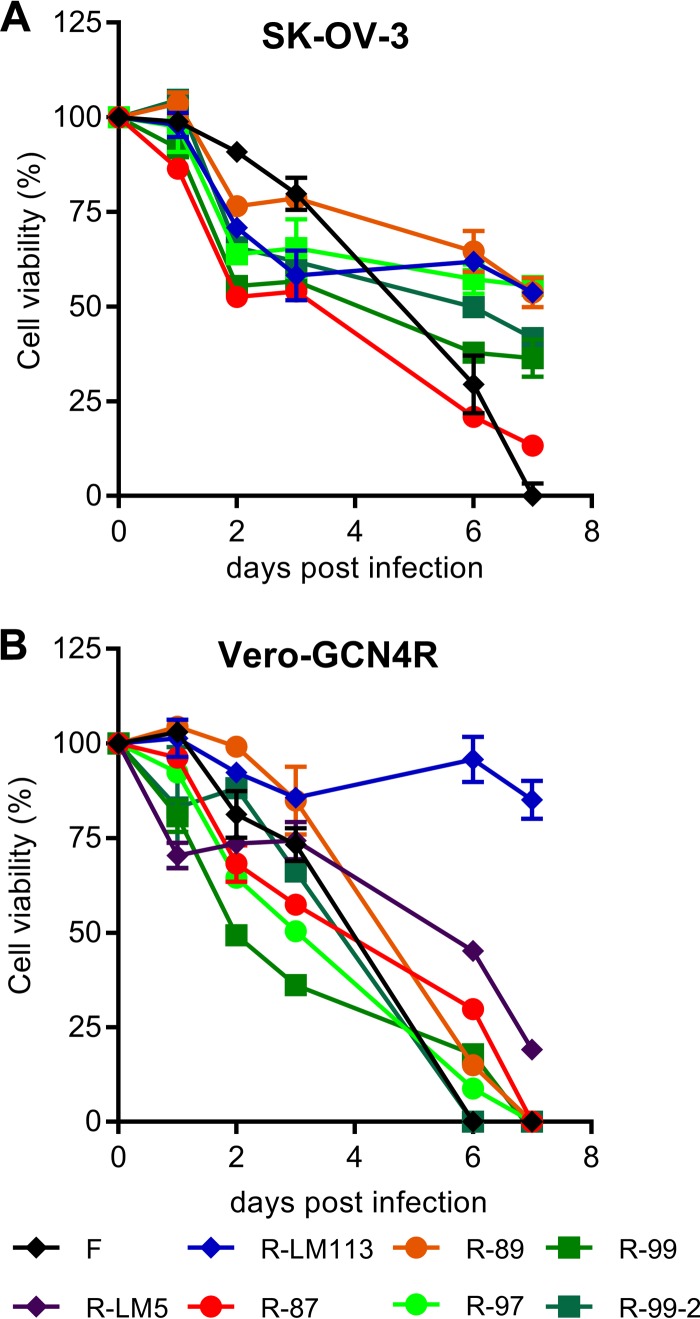
Cell-killing ability of the indicated viruses for SK-OV-3 and Vero-GCN4R cells. (A, B) SK-OV-3 (A) or Vero-GCN4R (B) cells were infected with the indicated recombinants or with HSV-1(F), R-LM5, or R-LM113 as controls, at 3 PFU/cell. Cell viability was quantified by the alamarBlue assay at the indicated days after infection. Results represent a typical experiment; each sample is the average from triplicate assays ± SD.

The above candidate oncolytic recombinants were tested for the ability to exert cytotoxic activity toward SK-OV-3 and Vero-GCN4R cells. Figure 6A shows that all recombinants were cytotoxic for SK-OV-3 cells. The highest effect was exhibited by the recombinants that replicated better. All the recombinants were cytotoxic also for Vero-GCN4R cells (Fig. 6B). As expected, the exception was R-LM113 in Vero-GCN4R cells, since the virus infects these cells at low efficiency. As noted earlier, the HER2-retargeted viruses infect Vero cells most likely through the simian HER2.

### Oncolytic efficacy of a double-gD-retargeted recombinant in immunocompetent mice.

**FIG 7:**
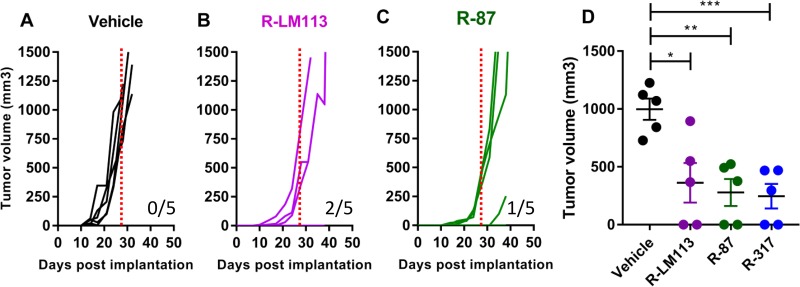
Antitumor activity of R-87. (A to C) Groups of 5 mice from the hHER2-transgenic C57BL/6 strain were implanted with hHER2-LLC-1 cells (0.2 × 10^6^ cell) in the left flank. Starting 3 days later, mice received four intratumoral treatments with the indicated viruses (1 × 10^8^ PFU/treatment), at 3 to 4 days' intervals. Tumor volumes for each treatment group are shown. (D) Distribution of the tumor size at 28 days after the initial treatment. Statistical significance was calculated using the *t* test: *, *P* < 0.05; **, *P* < 0.01; ***, *P* < 0.001. This experiment is the same as that shown in Fig. 6 of the companion paper (45).

We selected R-87, one of the best-performing double-gD-retargeted recombinants, to evaluate the oncolytic efficacy in immunocompetent mice. The animal model will be described elsewhere in detail under different coauthorship (V. Leoni, A. Vannini, V. Gatta, J. Rambaldi, M. Sanapo, C. Barboni, A. Zaghini, P. Nanni, P.-L. Lollini, C. Casiraghi, and G. Campadelli-Fiume, unpublished data). Essentially, it consists of the Lewis lung murine carcinoma 1 (LLC-1) cells made transgenic for human HER2 (hHER2-LLC-1). The cancer cells were implanted in a strain of the syngeneic C57BL/6 mice, which are transgenic for, and hence tolerant to, hHER2. Three days after implantation of the tumor cells, R-87 was administered intratumorally (i.t.) at 3 to 4 days' intervals, with 1 × 10^8^ PFU/injections, for a total of 4 treatments. As a comparison, we included in the experiment the prototypic R-LM113 and R-317 described in the companion paper (45). Figure 7A to C shows that the antitumor efficacy of R-87 was very similar to those of R-LM113 and of R-317, and the tumor size was significantly smaller than that in the untreated mice at 28 days (Fig. 7D).

## DISCUSSION

Recently, we developed a system for the cultivation in noncancer cells of clinical-grade oncolytic HSVs retargeted to HER2 and, potentially, to any cancer-specific receptor of choice (39). The potentially universal system is based on a double-retargeting strategy. One retargeting is to the cancer receptor, exemplified in our studies by HER2. The other retargeting is by way of the 20-aa-long GCN4 peptide, which readdresses the tropism to Vero cells expressing the artificial GCN4R. Here, we asked whether a double retargeting via gD is feasible and whether it can be optimized by means of a less disadvantageous detargeting strategy, designed on the structural analysis of the gD-nectin1 cocrystal (42). We report that gD simultaneously accepts two different heterologous ligands for retargeting to two different receptors. The double retargeting can be combined with novel nectin1-detargeting strategies, based on small deletions at two different loci in gD.

Analysis of the panel of gD recombinants shows that all of them were simultaneously retargeted to GCN4R and to HER2 and detargeted from both HVEM and nectin1. A novel finding to emerge from this investigation is that the modifications to the locus around aa 214 to 223 is suitable for nectin1 detargeting and retargeting. Each of the two heterologous receptors (HER2 and GCN4R) can be used alternatively to the other and independently of the other. The recombinants switched readily from one cell system (GCN4R-positive cell) to the other (HER2-positive cell).

Not all the insertion sites were equivalent, and the combination of ligand to insertion site can be optimized. This conclusion rests on the following examples.

A comparison of R-87 and R-97 shows that they share the following properties. They carry the same deletion in gD (aa 35 to 39) for nectin1 detargeting. They carry one of the two inserts (the 260-aa-long scFv to HER2 or the 20-aa-long GCN4 peptide) between aa 24 and 25 for HVEM detargeting. They differ in the relative positions of the two inserts. Thus, in R-87 the gD deletion is replaced by scFv, whereas in R-97 the deletion is replaced by the GCN4 peptide. R-87 and R-97 grew to very similar yields. Hence, exchanging the short GCN4 peptide and scFv at either one of these two positions was irrelevant with respect to growth capacity.

A comparison of R-89 and R-99 showed that they share the following properties. They carry the same deletion in gD (aa 214 to 223) for nectin1 detargeting. They carry one of the two inserts (scFv to HER2 or the GCN4 peptide) between aa 24 and 25 for HVEM detargeting. They differ in the relative positions of the two inserts. Thus, in R-89 the gD deletion is replaced by scFv, whereas in R-99 the deletion is replaced by the GCN4 peptide. The R-89 and R-99 recombinants replicated in a similar manner, and there was no apparent effect of the relative positions of the two inserts. Of note, the yields of R-89 and R-99 were lower than those of R-87 and R-97. Hence, a 10-aa deletion at this locus does not enable a highly efficient replication.

A comparison of R-97 and R-99-2 sheds light on the effects of performing the deletion in the aa 35 to 39 locus versus the 219 to 223 locus. At 48 h, these two recombinants replicated in a very similar manner in both SK-OV-3 and Vero-GCN4R cells, although a difference was seen at 24 h. Thus, a 5-aa deletion at either one of these two loci results in very similar recombinants.

Comparison of R-99 versus R-99-2 showed that these two recombinants differ in that R-99-2 carries a smaller (5-aa) deletion than R-99 (10-aa deletion) but are otherwise identical. The important result here is that R-99-2 grew 1 log more that R-99, suggesting that the size of the deletion may be critical for a better preservation of gD functions. This may explain why R-87, R-97, and R-99-2, which carry 5-aa-long deletions, replicated to similar yields. Of note, the differences in virus yield were not fully recapitulated in the plaque size; the latter is influenced not only by virus replication but also by the ability to perform cell-to-cell spread.

The R-87 recombinant was selected to evaluate the antitumor efficacy *in vivo*, in an immunocompetent mouse model. In general, the murine cancer cells are not as permissive to HSV as the human cancer cells; hence, this model underestimates the antitumor efficacy, a property shared with the vast majority of murine cancer models for oncolytic HSVs (46, 47). Here, the important result was that the antitumor efficacy of R-87 could not be differentiated from that of R-LM113 and of the gB recombinant R-317, described in the companion paper (45). Thus, the replication properties in cell cultures are recapitulated in the *in vivo* antitumor efficacy, and a recombinant carrying two retargeting moieties in gD is not at a disadvantage relative to R-LM113, which carries a single retargeting moiety. Altogether, the double retargeting via gD was feasible. By optimizing the detargeting strategies, we generated double-retargeted gD recombinants, which replicated as efficiently as the singly retargeted R-LM113 or the nondetargeted R-LM5, and which exerted antitumor activity *in vivo* as efficiently as R-LM113.

In a companion paper, we show that double retargeting is feasible also by insertion of the GCN4 peptide in gB and of the scFv in gD (45); even in that study (45), a novel gD detargeting strategy was developed. In both studies, the comparison of a number of recombinants led to optimization of double-retargeted recombinants. Together with the previous finding that the double retargeting is achieved by insertion of the GCN4 peptide in gH and of the scFv to HER2 in gD (39), current data indicate that several alternative strategies have become possible, now that we have increased the number of HSV glycoproteins that can serve as retargeting tools and in the light of accurate knowledge of gD-receptor structures (42). All in all, the double-gD recombinants and the gB/gD simultaneous retargeting yielded recombinants that replicate at comparable yields and will help move the field of retargeted oncolytic HSVs into the translational phase.

## MATERIALS AND METHODS

### Viruses.

R-LM5 and R-LM113 were described previously (17) (see Table 1 for a summary of genotypes and tropism). R-LM5 carries wt-gD open reading frame (ORF), the bacterial artificial chromosome (BAC) sequences cloned in the UL3-UL4 intergenic region, as in the parental pYeBac 102 (48), and the enhanced green fluorescent protein (EGFP) ORF under the α27 promoter cloned within the BAC sequences (17). It is not detargeted/retargeted; therefore, it is the wt counterpart of R-LM113, R-87, R-89, R-97, R-99, and R-99-2. R-LM113 is identical to R-LM5, except that it carries a HER2-retargeted gD. In particular, the deletion of aa 6 to 38 of mature gD, which removes critical residues for interaction with HVEM and nectin1 and its replacement with the scFv to HER2 derived from trastuzumab (44), detargets the virus tropism from the natural receptors. wt HSV-1 F was described previously (49).

### Engineering of R-87, R-89, R-97, R-99, and R-99-2.

These viruses are described in European patent application PCT/EP2017/063948 (Leoni and Campadelli, 14 December 2017). To engineer the gD double-retargeted recombinants, we constructed two precursor BACs, BAC 81 and BAC 91, starting from LM55 BG BAC. BAC 81 carries the GCN4 peptide between aa 24 and 25 of gD, whereas BAC 91 carries scFv HER2 in the same position. The HSV-1 recombinants R-87 and R-89 were derived from BAC 81 by insertion of the scFv HER2 in place of aa 35 to 39 (R-87) or in place of aa 214 to 223 (R-89). The recombinants R-97, R-99, and R-99-2 were derived from BAC 91 by insertion of the GCN4 peptide in place of aa 35 to 39 (R-97), in place of aa 214 to 223 (R-99), or in place of aa 219 to 223 (R-99-2) (Fig. 1B and Table 1). The amino acid sequence of the GCN4 peptide was KNYHLENEVARLKKLG. The core YHLENEVARLKK residues represent the epitope recognized by the single-chain antibody C11L34-H6 (PDB number 1P4B) (40). In the recombinant viruses, the GCN4 peptide was preceded and followed by GS linkers. The starting material for the engineering of BAC 81 and BAC 91 was LM55 BG BAC, which carries LOX-P-bracketed pBeloBAC11 and EGFP sequences inserted between U_L_3 and U_L_4 of HSV-1 genome (17). The engineering was performed in bacteria by means of *galK* recombineering, in two steps (38, 50). In the first step, the *galK* cassette, with homology arms to gD, was inserted between aa 24 and 25 of mature gD. In the second step, the *galK* insert was replaced with the GCN4 peptide cassette to generate the precursor BAC 81 or was replaced by the scFv HER2 cassette to generate the precursor BAC 91.

To carry out the first step in the engineering of BAC 81, the *galK* cassette, with homology arms to gD, was amplified by means of primers gD24_galK_f and gD25_galK_r (Table 2), using p-galK plasmid as the template. The PCR-amplified *galK* cassette was then electroporated into SW102 bacteria, which carry the LM55 BG BAC, to generate BAC 80. To exclude *galK* false-positive colonies, the recombinant clones were plated on MacConkey agar base plates, supplemented with 1% galactose and 12 μg/ml chloramphenicol, and checked by colony PCR. Colony PCR was carried out with primers galK_827_f and galK_1142_r (Table 2). To carry out the second step and generate the precursor BAC 81, a cassette encoding the GCN4 peptide (GenBank accession number AF416613.1) (40) bracketed by the downstream and upstream Gly-Ser linkers and by homology arms to gD was generated, through annealing and extension of the partially overlapping oligonucleotides gD24_GCN4_fB and gD25_GCN4_rB (Table 2). The oligonucleotides contained a silent BamHI restriction site, for screening purposes. The amplimer encoding the GCN4 cassette, with homology arms to gD, was electroporated into SW102 bacteria carrying BAC 80. The recombinant BAC was named BAC 81. Positive bacterial clones were checked by means of BamHI restriction analysis on colony PCR fragments, amplified with primers gD_ext_f and gD_ext_r (Table 2).

**TABLE 2:** Oligonucleotides employed to engineer the indicated recombinants

Recombinant and insertion, insertion location, or use	Oligonucleotide sense, name, and sequence
BAC-81, GCN4 peptide cassette inserted between aa 24 and 25 of mature gD of LM55 BAC	
*galK* insertion in gD 24–25	Forward, gD24_galK_f, CTC TCA AGA TGG CCG ACC CCA ATC GCT TTC GCG GCA AAG ACC TTC CGG TCC CTG TTG ACA ATT AAT CAT CGG CA
	Reverse, gD25_galK_r, TGG ATG TGG TAC ACG CGC CGG ACC CCC GGA GGG TCG GTC AGC TGG TCC AGT CAG CAC TGT CCT GCT CCT T
Colony PCR for screening	Forward, galK_827_f, GCG TGA TGT CAC CAT TGA AG
	Reverse, galK_1142_r, TAT TGT TCA GCG ACA GCT TG
GCN4 cassette insertion in place of *galK*	Forward, gD24_GCN4_fB, CTC TCA AGA TGG CCG ACC CCA ATC GCT TTC GCG GCA AAG ACC TTC CGG TCG GAT CCA AGA ACT ACC ACC TGG AGA ACG AGG TGG CCA GAC TGA AGA AGC TGG TGG GCA GC
	Reverse, gD25_GCN4_rB, TGG ATG TGG TAC ACG CGC CGG ACC CCC GGA GGG TCG GTC AGC TGG TCC AGG CTG CCC ACC AGC TTC TTC AGT CTG GCC ACC TCG TTC TCC AGG TGG TAG TTC TTG GAT CC
Colony PCR for screening	Forward, gD_ext_f, TCC ATA CCG ACC ACA CCG ACG AAT CCC
	Reverse, gD_ext_r, GAG TTT GAT ACC AGA CTG ACC GTG
R-87, scFv HER2 inserted in gD Δ35–39 of BAC 81	
*galK* insertion in gD Δ35–39	Forward, galK_gD35_F, TGA AGA AGC TGG TGG GCA GCC TGG ACC AGC TGA CCG ACC CTC CGG GGG TCC CTG TTG ACA ATT AAT CAT CGG CA
	Reverse, galK_gD39_R, GTG ATC GGG AGG CTG GGG GGC TGG AAC GGG TCT GGT AGG CCC GCC TGG ATT CAG CAC TGT CCT GCT CCT T
scFv HER2 insertion in place of *galK*	Forward, gD-34-scFvHER2-F, TGA AGA AGC TGG TGG GCA GCC TGG ACC AGC TGA CCG ACC CTC CGG GGG TCG AGA ATT CCG ATA TCC AGA T
	Reverse, gD-40-scFvHER2-R, GTG ATC GGG AGG CTG GGG GGC TGG AAC GGG TCT GGT AGG CCC GCC TGG ATG GAT CCA CCG GAA CCA GAG C
colony PCR for screening	Forward, gD_ext_f, TCC ATA CCG ACC ACA CCG ACG AAT CCC
	Reverse, scFv_456_r, AGC TGC ACA GGA CAA ACG GAG TGA GCC CCC
R-89, scFv HER2 inserted in gD Δ214–223 of BAC 81	
*galK* insertion in gD Δ214–223	Forward, galK_gD214_F, CCT ACC AGC AGG GGG TGA CGG TGG ACA GCA TCG GGA TGC TGC CCC GCT TCC CTG TTG ACA ATT AAT CAT CGG CA
	Reverse, galK_gD223_R, CTC GTG TAT GGG GCC TTG GGC CCG TGC CAC CCG GCG ATC TTC AAG CTG TAT CAG CAC TGT CCT GCT CCT T
scFv HER2 insertion in place of *galK*	Forward, gD213-scFvHER2f, CCT ACC AGC AGG GGG TGA CGG TGG ACA GCA TCG GGA TGC TGC CCC GCT TCG AGA ATT CCG ATA TCC AGA T
	Reverse, gD224-scFvHER2r, CTC GTG TAT GGG GCC TTG GGC CCG TGC CAC CCG GCG ATC TTC AAG CTG TAG GAT CCA CCG GAA CCA GAG C
Colony PCR for screening	Forward, gDintforw, CCC TAC AAC CTG ACC ATC GCT TGG
	Reverse, scFv_456_r, AGC TGC ACA GGA CAA ACG GAG TGA GCC CCC
BAC 91, scFv HER2 cassette inserted between aa 24 and 25 of mature gD of LM55 BAC	
scFv HER2 insertion in place of *galK*	Forward, gD24-scFvHer2-F, CTC TCA AGA TGG CCG ACC CCA ATC GCT TTC GCG GCA AAG ACC TTC CGG TCG AGA ATT CCG ATA TCC AGA TG
	Reverse, gD25-scFvHer2-R, TGG ATG TGG TAC ACG CGC CGG ACC CCC GGA GGG TCG GTC AGC TGG TCC AGG GAT CCA CCG GAA CCA GAG C
Colony PCR for screening	Forward, gD_ext_f, TCC ATA CCG ACC ACA CCG ACG AAT CCC
	Reverse, scFv_456_r, AGC TGC ACA GGA CAA ACG GAG TGA GCC CCC
R-97, GCN4 inserted in gD Δ35–39 of BAC 91	
*galK* insertion in gD Δ35–39	Forward, gD35-galK-F, GCT CTG GTT CCG GTg GaT CCC TGG ACC AGC TGA CCG ACC CTC CGG GGG TCC CTG TTG ACA ATT AAT CAT CGG CA
	Reverse, gD39-galK-R, GTG ATC GGG AGG CTG GGG GGC TGG AAC GGG TCT GGT AGG CCC GCC TGG ATT CAG CAC TGT CCT GCT CCT T
GCN4 insertion in place of *galK*	Forward, gD35-GCN4-F, GCT CTG GTT CCG GTg GaT CCC TGG ACC AGC TGA CCG ACC CTC CGG GGG TCG GAT CCA AGA ACT ACC ACC TGG AGA ACG AGG TGG CCA GAC TGA AGA AGC TGG TGG GCA GC
	Reverse, gD39-GCN4-R, GTG ATC GGG AGG CTG GGG GGC TGG AAC GGG TCT GGT AGG CCC GCC TGG ATG CTG CCC ACC AGC TTC TTC AGT CTG GCC ACC TCG TTC TCC AGG TGG TAG TTC TTG GAT CC
Colony PCR for screening	Forward, scFv4D5 651_f, GGA CAC TGC CGT CTA TTA TTG TAG CCG CT
	Reverse, gDintrev, CCA GTC GTT TAT CTT CAC GAG CCG
R-99, GCN4 inserted in gD Δ214–223 of BAC 91	
*galK* insertion in gD Δ214–223	Forward, galK_gD214_F, CCT ACC AGC AGG GGG TGA CGG TGG ACA GCA TCG GGA TGC TGC CCC GCT TCC CTG TTG ACA ATT AAT CAT CGG CA
	Reverse, galK_gD223_R, CTC GTG TAT GGG GCC TTG GGC CCG TGC CAC CCG GCG ATC TTC AAG CTG TAT CAG CAC TGT CCT GCT CCT T
GCN4 insertion in place of *galK*	Forward, gD213-GCN4-F, CCT ACC AGC AGG GGG TGA CGG TGG ACA GCA TCG GGA TGC TGC CCC GCT TCG GAT CCA AGA ACT ACC ACC TGG AGA ACG AGG TGG CCA GAC TGA AGA AGC TGG TGG GCA GC
	Reverse, gD224-GCN4-R, CTC GTG TAT GGG GCC TTG GGC CCG TGC CAC CCG GCG ATC TTC AAG CTG TAG CTG CCC ACC AGC TTC TTC AGT CTG GCC ACC TCG TTC TCC AGG TGG TAG TTC TTG GAT CC
Colony PCR for screening	Forward, gDintforw, CCC TAC AAC CTG ACC ATC GCT TGG
	Reverse, HSV_139688_r, CCG ACT TAT CGA CTG TCC ACC TTT CCC
R-99-2, GCN4 inserted in gD Δ219–223 of BAC 91	
*galK* insertion in gD Δ214–223	Forward, galK_gD214_F, CCT ACC AGC AGG GGG TGA CGG TGG ACA GCA TCG GGA TGC TGC CCC GCT TCC CTG TTG ACA ATT AAT CAT CGG CA
	Reverse, galK_gD223_R, CTC GTG TAT GGG GCC TTG GGC CCG TGC CAC CCG GCG ATC TTC AAG CTG TAT CAG CAC TGT CCT GCT CCT T
GCN4 insertion in place of *galK*	Forward, gD219-GCN4-F, CCT ACC AGC AGG GGG TGA CGG TGG ACA GCA TCG GGA TGC TGC CCC GCT TCA TCC CCG AGA ACC AGC GCG GAT CCA AGA ACT ACC ACC TGG AGA ACG AGG TGG CCA GAC TGA AGA AGC TGG
	Reverse, gD224-GCN4-R, CTC GTG TAT GGG GCC TTG GGC CCG TGC CAC CCG GCG ATC TTC AAG CTG TAG CTG CCC ACC AGC TTC TTC AGT CTG GCC ACC TCG TTC TCC AGG TGG TAG TTC TTG GAT CC
Colony PCR for screening	Forward, gDintforw, CCC TAC AAC CTG ACC ATC GCT TGG
	Reverse, HSV_139688_r, CCG ACT TAT CGA CTG TCC ACC TTT CCC

The precursor BAC 91 carries scFv to HER2 between aa 24 to 25 of gD. It was generated from BAC 80. First, the scFv HER2 cassette bracketed by homology arms to gD was amplified by means of primers gD24-scFvHer2-F and gD25-scFvHer2-R (Table 2). Bacterial colonies were checked for the presence of the sequence of choice by means of colony PCR with primers gD_ext_f and scFv_456_r (Table 2).

To engineer R-87 and R-89, the scFv HER2 was inserted in gD Δ35–39 (R-87), or in gD Δ214–223 (R-89), as detailed for BAC 81, by means of oligonucleotides reported in Table 2. To engineer R-97, R-99, and R-99-2, the GCN4 peptide was inserted in gD Δ35–39 (R-97), gD Δ214–223 (R-99), or gD Δ219–223 (R-99-2) of BAC 91, by means of the oligonucleotides reported in Table 2.

To reconstitute the recombinant viruses, 500 ng of recombinant BAC DNA was transfected into the Vero-GCN4R cell line and the SK-OV-3 cell line by means of Lipofectamine 2000 (Life Technologies) and then grown in these cells. Virus growth was monitored by green fluorescence. The structure of the recombinant was verified by sequencing the entire gD. Virus stocks were generated in Vero-GCN4R and SK-OV-3 and titrated in Vero-GCN4R and SK-OV-3 cells. All other recombinants were engineered by the same procedure, by means of the oligonucleotides described in Table 2.

### Tropism of R-87, R-89, R-97, R-99, and R-99-2.

The indicated cells were infected with the indicated viruses at 1 PFU/cell and monitored 24 h later with a Nikon Eclipse TS100 fluorescence microscope. Where indicated, infection was carried out in the presence of the MAb to HER2 (trastuzumab) at a concentration of 28 μg/ml.

### Determination of virus growth and extent of viral progeny release.

Vero-GCN4R and SK-OV-3 cells were infected with wt HSV-1 F, R-LM5, R-LM113, R-87, R-89, R-97, R-99, and R-99-2 at 0.1 PFU/cell. Unabsorbed virus was inactivated by rinsing the cells with a pH 3 solution (40 mM citric acid, 10 mM KCl, 135 mM NaCl). Replicate cultures were frozen at 24 and 48 h after infection. Progeny virus (intracellular plus extracellular) was titrated in SK-OV-3 cells. Results are expressed as the means from three independent experiments ± standard deviations (SD). In virus release experiments, replicate cultures of Vero-GCN4R or SK-OV-3 infected with R-LM113, R-87, or R-89 at 0.1 PFU/cell were harvested 48 h after infection as cell lysates plus medium. Alternatively, medium or cellular fractions were harvested separately. Progeny virus was titrated in SK-OV-3 cells. Results are expressed as the means from three independent experiments ± SD.

### Plating efficiency and relative plaque size.

Replicate aliquots of R-LM5, R-LM113, R-87, R-89, R-97, R-99, and R-99-2 were plated on Vero-GCN4R and SK-OV-3 cells, and the plaques were counted 3 days later. Results represent the means from three independent infections ± SD. For plaque size determination, pictures of 6 individual plaques from each of the above samples were taken 3 days after infection. Plaque areas were measured with Nis Elements-Imaging Software (Nikon). Each result represents the mean area ± SD.

### Cytotoxicity assay.

SK-OV-3 and Vero-GCN4R cells were seeded in 96-well plates (8 × 10^3^ cell/well) and infected with wt HSV-1 F, R-LM5, R-LM113, R-87, R-89, R-97, R-99, and R-99-2 (3 PFU/cell) or mock infected. alamarBlue (10 μl/well; Life Technologies) was added to the culture media at indicated times after infection, and the cultures were incubated for 4 h at 37°C. Plates were read at 560 and 600 nm with the GloMax Discover system (Promega Corporation). For each time point, cell viability was expressed as the percentage of alamarBlue reduction in infected versus uninfected cells, after subtraction of the background value (medium alone). Each point represents the average from at least triplicate samples ± SD.

### *In vivo* antitumor efficacy.

C57BL/6 mice transgenic for and tolerant to hHER2, received from Jackson Laboratories, were implanted with the murine Lewis lung carcinoma 1 (LLC-1) cells made transgenic for hHER2 (hHER2-LLC-1), 0.2 × 10^6^ cells/mouse (Leoni et al., unpublished). Three days later, mice received R-87, R-LM113, and R-317 as control viruses, peri-intratumorally (i.t.), four dosages/mouse at 3 to 4 days' intervals (1 × 10^8^ PFU/injection); there were 5 mice for each treatment group. Tumor size was measured by means of a caliper at the indicated days as described previously (19). Animal experiments were performed according to European directive 2010/63/UE, Italian laws 116/92 and 26/2014. Experimental protocols were reviewed and approved by the University of Bologna Animal Care and Use Committee (“Comitato per il Benessere degli Animali, COBA”) and approved by the Italian Ministry of Health, Authorization number 86/2017-PR to Anna Zaghini.
